# British Institutions for the Care of the Inebriate.—VIII

**Published:** 1904-04-02

**Authors:** 


### HOSPITAL ADMINISTRATION.

### CONSTRUCTION AND ECONOMICS.

### BRITISH INSTITUTIONS FOR THE CARE OF THE INEBRIATE.—VIII.

By Our Own Medical Commissioner.

#### HANCOX HOME FOR MALE INEBRIATES.

The institutional treatment of inebriety is still in the experimental stage. With the coming of clearer views as to its pathology, obsolete methods and mediæval procedures are slowly giving way and blind empiricism is being replaced by a science-directed rational management. Innumerable difficulties surround the problem. Ethical and legal considerations must receive due attention. We are still far from unanimity even in matters medical. All investigations scientifically designed and carried out in accordance with the dictates of common sense backed up by reliable clinical experience are to be welcomed. As we have had occasion to point out in former articles not a few of the private retreats now in existence are conducted primarily for the pecuniary advantage of the proprietors. The time is rapidly drawing near when the Inebriates Acts will have to be so amended that registration is made compulsory and every establishment is thrown open to H. M Inspector. Meanwhile the institutions which have been founded by philanthropic enterprise deserve encouragement. Many of these are accomplishing much good work, often under circumstances of peculiar difficulty. Foremost among the bodies which have undertaken the care of inebriates is the Church of England Temperance Society, of which F. Eardley-Wilmot, Esq., of 4 The Sanctuary, Westminster, S.W. is the General Secretary. We hope to deal individually with the various homes conducted by this Society, and in the present article we shall treat only of the Home for Male Inebriates.

The Hancox Home is situated in a very charming district of Sussex, some three and a half miles from Battle and Robertsbridge stations on the South Eastern and Chatham Railway. It is about seven miles from Hastings. The nearest village is Whatlington. The estate consists of eight acres, well placed on high ground with attractive views of undulating and well-wooded country. The property is leasehold. In 1900 the house and farm were offered rent free, and a considerable amount has been expended in adapting and extending the premises for the present purpose. The house is a rambling, ancient one, and by no means an ideal residence for inebriates. Many of the rooms are small and some are low, and the ventilation is necessarily imperfect and the sanitation might with advantage be improved. The dormitories at the time of our visit were much overcrowded and the accommodation for clothes meagre. The best room in the house, situated on the ground floor, is devoted to the purposes of a chapel. We would suggest, with all respect and reverence, that the purposes of true religion might be more perfectly served if the chapel were converted into a dormitory, or at least allowed to be used also as a reading-room.

The Home is licensed, under the Act of 1879, for 20 patients, but at the time of our visit there were 40 inmates.

Arrangements are provided for two classes of cases. The so-called first-class pay 15s. a week, have separate dining-room, superior sleeping accommodation and engage chiefly in gardening and light work; the second-class pay 9s., a week have a common dining-room, poorer dormitories, engage in the work of the house, and heavy labour in the fields.

It must at once be admitted that the fees charged are exceptionally low. It is much to be hoped that only suitable cases are admitted at these cheap rates. We understand a number of clergymen have undergone treatment at Hancox. We are strongly of opinion that for the sake of all parties it is most unwise to allow the clergy admittance to such a home as this. As is well known the available homes for male inebriates where they can receive satisfactory treatment at low rates are exceedingly small, indeed Hancox is practically the only one conducted by a public body, and we therefore consider it essential that it should be strictly reserved for those who cannot pay or whose friends cannot provide the fees charged by more expensive establishments.

Hancox must be studied as an interesting experiment. Work is rightly considered an essential element in treatment. Everyone is supposed to engage for eight hours daily in work. We are inclined to think it would be wise to insist that smoking should not be permitted during working hours. Out-door work is particularly encouraged. The soil is fairly good and is well cultivated. The orchard, garden, and poultry farm offer plenty of opportunities for work. A large number of pigs are kept. The workshops might well be enlarged, and it would be a great advantage if more skilled instruction could be provided.

Much insistance is made on the religious services, attendance at which is compulsory. Morning and evening prayers are conducted by the superintendent. Two sermons are given on each Sunday. Holy Communion is celebrated fortnightly, and every Friday a Bible class is held. The Vicar of Whatlington acts as chaplain. The home is directed in accordance with Church of England procedures, but we understand members of other communions may be admitted.

The committee consists of the Rev. Prebendary Pennethorne, the Rev. H. Russell Wakefield, the Rev. E. Grose Hodge, and the Hon. A. Holland Hibbert. The secretary is F. Eardley-Wilmot, Esq. The house committee is composed of the following:—The Very Rev. the Dean of Battle, the Rev. C. W. G. Wilson, the Rev. E. J. Sing, W. H. Mullins, Esq., J.P., H. T. B. Combe, Esq., J.P., and E. Sheppard, Esq. The chaplain is the Rev. S. F. Hiron. B. E. Gott acts as superintendent. Mr. Gott and his wife are responsible for the conduct of the Home. No women, with the exception of the cook who is the wife of the first officer, are employed.

The Home was only opened in November of 1900, so that it is hardly fair to demand results as yet. According to the latest available records* it was shown that "on December 31st, 1901, there were 32 patients in the Home. During the year there were 51 patients admitted, and 48 were discharged, leaving 35 patients in the Home on December 31st, 1902."

Unfortunately, at the time of our visit the day was exceedingly wet, and we were unable to inspect the men at their work in the open. We were much struck, however, with the facilities available for useful employment in the field. We noted that there was an excellent bowling green,

* "The Report of the Inspector under the Inebriates Acts, 1879 to 1900. For the year 1902." London: Eyre and Spottiswoode September 1903.

which, however, needed draining, for it appeared much water-logged, and a very good cricket pitch. The indoor accommodation, it must be admitted, was somewhat disappointing. The recreation and reading rooms might be improved with advantage. The earth closets should receive more careful attention. The water supply is obtained from a well on the estate. If the funds were only forthcoming much might readily be done to improve both house and grounds, and increase the comfort of the inmates, and so further the success of treatment. We understand that the institution will probably be removed to more convenient premises, as it is proposed to perpetuate the memory of the late Archbishop Temple by placing the work now being carried on at Hancox on a more satisfactory and permanent basis. Certainly the work stands in need of assistance. In spite of limited means, and some considerable difficulties arising from many causes, much good work is evidently being accomplished. The work may be commended to practical temperance reformers.

Those interested in the conduct of such homes will be interested in studying the following table:—

Winter Routine.

*First-Class Patients.*

| | | |
|---|---|---|
| 7.30 | A.M. | Hour of rising. |
| 8.0 | „ | Prayers in chapel. |
| 8.15 | „ | Breakfast. |
| 9.0 | „ | Work in garden or workshops. |
| 12.30 | P.M. | Work ceases. Prepare for dinner. |
| 1.0 | „ | Dinner. |
| 2 30 | „ | Work in garden or workshops. |
| 4.30 | „ | Work ceases (earlier if dark). |
| 5.30 | „ | Tea. After tea, recreation in house and recreation room. |
| 9.0 | „ | Prayers in chapel. |
| 9.15 | „ | Supper. |
| 10.0 | „ | Lights out downstairs. |
| 10.30 | „ | Lights out upstairs. |

*Second-Class Patients.*

| | | |
|---|---|---|
| 7.0 | A.M. | Hour of rising. |
| 7.30 | „ | Breakfast. |
| 8.0 | „ | Prayers in chapel. |
| 8.30 | „ | Work in field or workshops as arranged. House Orderly, No. 1.—To sweep and clean bedrooms and staircases, turn down bedding for airing. House Orderly, No. 2.—To sweep and clean lower portion of house, assist in kitchen, clean knives, etc. |
| 12.0 | noon. | Work ceases. |
| 12.30 | P.M. | Dinner. |
| 2.0 | „ | Work in field or workshops as arranged. House Orderly, No. 1.—Make beds. House Orderly, No. 2.—Assist in kitchen. |
| 4.30 | „ | Work ceases (earlier if dark). |
| 5.0 | „ | Tea. After tea, recreation in house and recreation room. |
| 9.0 | „ | Prayers in chapel. |
| 9.15 | „ | Supper. |
| 9.45 | „ | Lights out downstairs. |
| 10.15 | „ | Lights out upstairs. |

Those desiring to send cases to Hancox would do well to study the following rules:—

I.—Application for admission to the Home to be made ONLY to the Secretary, F. Eardley-Wilmot, 4 The Sanctuary, Westminster, S.W.

II.—Agreements must be duly filled up by some friend, or friends, of the patient.

III.—The length of stay of each inmate must be at the discretion of the Resident Superintendent and Secretary. A residence of at least nine months is generally necessary.

IV.—Each inmate must submit to all rules and regulations, and fulfil without question whatever duties may be assigned to him by the Superintendent.

V.—In case of gross insubordination or misconduct, the Superintendent, in consultation with the Secretary, shall have power to eject the patient.

VI.—In case of any inmate being seriously ill, the Hon. Physician will call upon the friends to remove him if in a fit state for removal.

VII.—No inmate may retain possession of money or valuables while in the Home.

VIII.—Inmates can only see their friends on the regular visiting days, except by special permission of the Superintendent.

IX.—Visitors must give into the keeping of the Superintendent any article or gifts brought on visiting days for the inmates. It must be understood that only such gifts as are approved will be given to the inmates.

X.—All letters, written, or received, to be left open for perusal by the Superintendent.

XI.—Inmates must bring suitable clothing. A list of articles required will be supplied.

XII.—Any special medicine required will be charged for.

Dr. Watkin W. Jones, of St. Leonards, attends weekly and when required. Cases ineligible for admission are those suffering from the following diseases:—(1) Fits of any kind; (2) insanity or imbecility; (3) cancer; (4) open wounds and sores; (5) consumption or other serious ailments requiring hospital treatment; (6) any other affection that may in the opinion of the honorary medical officers render the applicant unlikely to benefit by treatment. We learn that efforts are made to secure an effectual "after-care" of the patients when they leave the Home and suitable situations are often obtained for them.

With inceased financial support, wise management, effective medical control, and a sympathetic but scientific conduct of all departments of the work, the good services of Hancox should be much extended.

